# Obstetrics

**Published:** 1866-02

**Authors:** 


### OBSTETRICS.

*On the Propriety of Inducing Premature Delivery.* By T. G. Thomas, M. D., Professor of Obstetrics and the Diseases of Women and Children, in the College of Physicians and Surgeons, New York.

[Read before the N. Y. Obstetric Society.]

On the 1st of June, 1865, Mrs. B., a short but well made woman, aged thirty-three, applied to me to take charge of her delivery, which was anticipated about the 5th of September, giving me the following history. She had been delivered ten months before of a still-born child at the seventh month, labor being ushered in by a violent convulsion which destroyed the life of the child, and left the mother in so precarious a condition that her life for several days was despaired of. During her illness she was attended by an intelligent practitioner of Saratoga, and Dr. Willard Parker, of New York, in consultation. After recovery from this confinement, she was amaurotic for three months, and was during that time supposed to be suffering from Bright's Disease. Becoming again pregnant, she naturally dreaded a recurrence of convulsions, and fears of a fatal issue for herself and offspring filled her mind with anxious forebodings—more especially as one of her sisters, an unmarried lady, had since that time died of Bright's Disease.

Upon taking charge of the case, I made a careful chemical and microscopical examination of the urine, and found it free from evidences of disease. These examinations were repeated weekly with the same result; and as I left the city on the 1st of July, to be absent a month, I left my patient under the care of Dr. Charles A. Budd, who watched the secretion as I had done, and reported her doing well on the 1st of August. On the 12th of that month, I was called hastily to Mrs. B., and found her laboring under some dyspnœa, considerable confusion of intellect, dizziness of head and nausea. Œdema of the feet, hands and neck was discovered, and upon examining the urine, which was scanty and high colored, I found it loaded with albumen. After this I saw her daily, and adopted those measures most relied on in such cases as preventive of the threatening climax. From day to day the symptoms above recorded steadily increased, the œdema of the neck becoming so marked as to be oppressive, and great depression of spirits superadding itself to the array of morbid signs. On the 16th, Dr. Thebaud saw her with me in consultation, agreed with me in the propriety of inducing premature delivery for prevention of convulsions, which, from the peculiarly marked features of the case, we regarded as more than probable; and in accordance with the result of the consultation, I proceeded to accomplish it on the 17th.

On this date, at 1 P. M., I introduced into the cervix a large sponge tent, but finding it impossible to retain it in position I removed it and used the smallest of Barnes' dilators. After twenty-five minutes I had dilated the os to the size of a circle whose diameter is two inches. I now left her, and returned to her at 4 P. M. No labor pains having been excited, I introduced the largest dilator, and after dilating to its utmost capacity, introduced a No. 10 gum elastic catheter between the membranes and uterine wall, and left it. At 7 P. M., I was sent for in haste, violent expulsive pains being established. Upon my arrival the desired process was found fully inaugurated; the lady, as a further preventive means, was put under chloroform, and at $11\frac{1}{2}$ P. M. a female child was born.

Mother and child did well, no untoward symptom showing itself in the case of either.

My object in relating this case is to call out the views of members of the Society upon a point which I consider one of the most important and momentous in the whole field of obstetric practice; and before closing I desire to give, as concisely as I may, the reasons which actuated me in interfering in the case which I have brought to your notice.

First, let me state that I am fully aware of the fact, so patent to all, that a large proportion of women affected by puerperal albuminuria pass through labor without untoward symptoms, and rapidly recover after its accomplishment, and that I feel that I would be recording a mischievous and dangerous precedent did I by this history lead any one to interfere in the progress of utero-gestation merely on account of the development of this condition.

Albuminuria in the pregnant woman, then, let me premise, is insufficient as a ground on which to base premature delivery. In the case under consideration I was influenced and decided in my course by the following considerations:

1st. The patient's previous delivery and narrow escape from death from this very cause.

2d. The fact that even in the first month of her existing pregnancy she had given evidences of Bright's Disease.

3d. The sudden appearance of grave cerebral symptoms coincidently with marked, even excessive amount of albumen in the urine, and, what I omitted to mention, tube casts filled with granular epithelium.

Believing now, (after having carefully reflected upon the case,) as I then did, that I was saving my two patients from a great danger by incurring a comparatively slight one, I should again, if similarly circumstanced, act as I did.—*New York Medical Journal.*

